# Lessons Learnt From the First Year of a First-of-Its-Kind Breast Cancer Patient Psychosocial Support Group in Ethiopia and Promising Practices for Initiation and Implementation of Survivor Support Programs in Low-Resource Settings

**DOI:** 10.1200/GO.22.14000

**Published:** 2022-05-05

**Authors:** Biniyam Tefera Deressa, Etsegenet Bekele, Rahima Hussen, Breanne Lott

**Affiliations:** ^1^ Adama Hospital Medical College, Addis Ababa, Ethiopia; ^2^ The University of Arizona, Tucson, AZ

## Abstract

Psychosocial support helps cancer patients adjust to diagnosis and treatment and improve their overall well-being. With few resources committed to cancer prevention and treatment in Ethiopia, patients’ and survivors’ mental health needs are frequently disregarded, resulting in low quality of life. In October 2020, a support group for breast cancer (BC) patients was formed at Adama Hospital Medical College (AHMC), the first of its kind in Ethiopia. Here, we reflect on lessons learnt from the first year of implementation and share promising practices for establishing such groups in low-resource settings.

**METHODS:**

The founder and facilitators of the AHMC BC patient and survivor support group describe the program and critically reflect on its impact, challenges to implementation, lessons learned, and ideas for further improvement. Ng and Colombani’s framework for selecting best practices in public health (2015) is used to organize key themes.

**RESULTS:**

One Saturday each month, 20-30 patients and survivors gather with a traditional Ethiopian coffee ceremony. Participants report feelings of empowerment, hope, and confidence, a stronger sense of interconnectedness with others, and increased engagement in social activities. One notable success was the advocacy opportunity for the women to speak with hospital administrators and Ministry of Health officials at a BC Awareness Month celebration this year. Implementation challenges include a lack of funding, lack of a trained psychologist, and ethical uncertainty about distress caused when members die (not uncommon as patients typically present with advanced stage disease). Clinicians have learned important lessons about patients’ lay beliefs and consequently, patient counseling has been improved to incorporate topics of diet, sexuality, and pregnancy.

**CONCLUSION:**

A first-of-its-kind BC patient and survivor support group improves perceived psychosocial well-being and quality of life for women in Ethiopia. The low-cost program shares promising practices for survivor support groups and patient-centered cancer treatment in low-resource contexts.

